# Improving health system readiness to address violence against women and girls: a conceptual framework

**DOI:** 10.1186/s12913-022-08826-1

**Published:** 2022-11-28

**Authors:** Manuela Colombini, Susannah H. Mayhew, Claudia García-Moreno, Ana Flavia d’Oliveira, Gene Feder, Loraine J. Bacchus

**Affiliations:** 1 London School of Hygiene and Tropical Medicine, London, UK; 2 UNDP-UNFPA-UNICEF-WHO-World Bank Special Programme of Research, Development and Research Training in Human Reproduction (HRP), Department of Sexual and Reproductive Health and Research, World Health Organization, Geneva, Switzerland; 3 Sao Paulo University, Sao Paulo, Brazil; 4 Centre for Academic Primary Care, Bristol Medical School, University of Bristol, Bristol, UK

**Keywords:** Intimate partner violence, Health system readiness, Violence against women, Conceptual framework

## Abstract

**Background:**

There is an increasing focus on readiness of health systems to respond to survivors of violence against women (VAW), a global human rights violation damaging women’s health. Health system readiness focuses on how prepared healthcare systems and institutions, including providers and potential users, are to adopt changes brought about by the integration of VAW care into services. In VAW research, such assessment is often limited to individual provider readiness or facility-level factors that need to be strengthened, with less attention to health system dimensions. The paper presents a framework for health system readiness assessment to improve quality of care for intimate partner violence (IPV), which was tested in Brazil and Palestinian territories (oPT).

**Methods:**

Data synthesis of primary data from 43 qualitative interviews with healthcare providers and health managers in Brazil and oPT to explore readiness in health systems.

**Results:**

The application of the framework showed that it had significant added value in capturing system capabilities - beyond the availability of material and technical capacity - to encompass stakeholder values, confidence, motivation and connection with clients and communities. Our analysis highlighted two missing elements within the initial framework: client and community engagement and gender equality issues. Subsequently, the framework was finalised and organised around three levels of analysis: macro, meso and micro. The micro level highlighted the need to also consider how the system can sustainably involve and interact with clients (women) and communities to ensure and promote readiness for integrating (and participating in) change. Addressing cultural and gender norms around IPV and enhancing support and commitment from health managers was also shown to be necessary for a health system environment that enables the integration of IPV care.

**Conclusion:**

The proposed framework helps identify a) system capabilities and pre-conditions for system readiness; b) system changes required for delivering quality care for IPV; and c) connections between and across system levels and capabilities.

## Background

Violence against women (VAW) is a global human rights violation affecting at least 1 in 3 women globally [1], with adverse health, social, and economic consequences [2]. International organisations have called upon countries to develop health system responses to prevent and respond to such violence [3]. This call became even more urgent during the COVID-19 pandemic that saw a rise in reports of VAW [4–6].

Intimate partner violence (IPV) - one of the most prevalent forms of VAW - refers to behaviour by a current or past intimate partner that causes physical, sexual or psychological harm [7]. It is associated with interacting individual, interpersonal, community and structural and social/cultural factors, and underpinned by gender inequality. To overcome some of its adverse health effects, survivors tend to use health services at an increased rate [8, 9] than non-abused women. Furthermore, despite studies suggesting that health services are the first entry points for disclosure of such violence [7], the health systems response to IPV has lagged behind [9]. It follows that the interventions designed to respond to women who are affected by such violence are also complex and require change at multiple levels [9]. This includes the adoption of systemic approaches to integration and implementation of services which enable exploration of the many factors affecting effective service delivery [8, 10]. The integration of an IPV response within healthcare requires establishing procedures and building specific skills (such as empathic listening, validation and assisting with referrals to other services) that may require more time than is allocated per patient [11]. It also requires a change in the institutional medical culture (from ‘treat’ to ‘care and support’) [10] and in the structural factors (from normalisation to unacceptance) [12]. Furthermore, when new programmes to improve response to VAW are implemented, it is important to first assess the readiness of the receiving health system [10, 13]. However, limited information on the impact of integration and the necessary pre-conditions for achieving it continue to hamper successful integration of IPV into existing services and programmes [9, 14]. The integration of IPV care should be supported by a thorough assessment of the readiness of health systems. This should provide insight into which key services, procedures and resources (including trained/skilled providers) should be in place for a health care facility to offer care to survivors of violence [7, 15].

### Conceptualising health system readiness for integrating services and interventions

There is increasing focus on health system readiness to respond to survivors of violence, to identify gaps and better understand why some approaches to integration may be effective in one context, but not in others [13, 14] and how they affect already overstretched health systems. Existing evidence underlines the importance of organisational and individual readiness to support the implementation of interventions to address IPV in health systems [13]. Health system readiness has been used in various health contexts to refer to either a) providers’ ability to respond to a specific health issue (e.g. knowledge, motivation, attitudes) [10, 16]; b) services’ ability to assess availability, performance and quality of care offered (e.g. availability of services, infrastructures, supplies) [17]; or c) organizational readiness to implement a particular innovation (e.g. supportive environment, governance) [18]. Health system readiness focuses on how prepared healthcare systems and institutions - including providers and potential users - are to accept and implement the changes brought about by the integration of the new programme. In IPV research, such assessment is often limited to individual provider readiness [16, 19] or facility-level (or service-level) factors that need to be strengthened [15] with less attention – until recently [10, 20] - to broader health system dimensions (e.g. organisational issues such as governance and leadership) and their interactions [16, 21], which are harder to change. Two recent reviews on health providers’ barriers to address IPV highlight the importance of addressing organisational and structural factors for ensuring a supportive health system [10, 12].

The current literature on IPV and readiness has focused primarily on its measurement and specific tools were designed for this purpose. *Provider readiness* scales yield information on provider knowledge, self-efficacy, motivational and emotional readiness to respond to IPV, and are often used to assess training interventions. They fail, however, to consider contextual and health system factors that are beyond the control of the providers. To date, *service readiness* scholarship has focused on providing lists of indicators for service readiness inputs (e.g. availability and adequacy of infrastructure, supplies and resources), but does not offer a comprehensive framework for assessing health facilities’ capacity and preparedness to deliver and/or assimilate new services [17]. *Organisational readiness* for change scales have been used to assess readiness of healthcare organisations [22, 23] encompassing key domains such as staff motivation for change, resources, staff attributes and organisational climate (e.g., incentives, support, mentoring). However, most of these tools have failed to adopt a systemic approach to readiness that encompasses capabilities beyond providers’ preparedness and material and technical resources to include policy, leadership and governance issues, and clients’ and community awareness and engagement. Other limitations across these tools and measures include the lack of a cross-sector lens on health sector coordination of care with other services (e.g., police, legal services, non-governmental organisations and social services). They also do not pay attention to client and community engagement, which is an important and globally recognised strategy for advancing acceptability, demand, uptake and access to integrated care [24, 25] and for ensuring that health systems are better aligned to individuals’ and communities’ practices and needs [26]. Engagement is seen as an enabler in sexual and reproductive health integration to ensure client and community preparedness [25, 27]. Yet, community engagement is often overlooked in the health system response to IPV.

In this article we present a framework for health system readiness assessment to improve quality care for IPV [9, 15], which was tested and refined in Brazil and Palestine. This also lays the groundwork for developing further measures of readiness to integrate IPV services.

## Conceptual framework

**Fig. 1:**
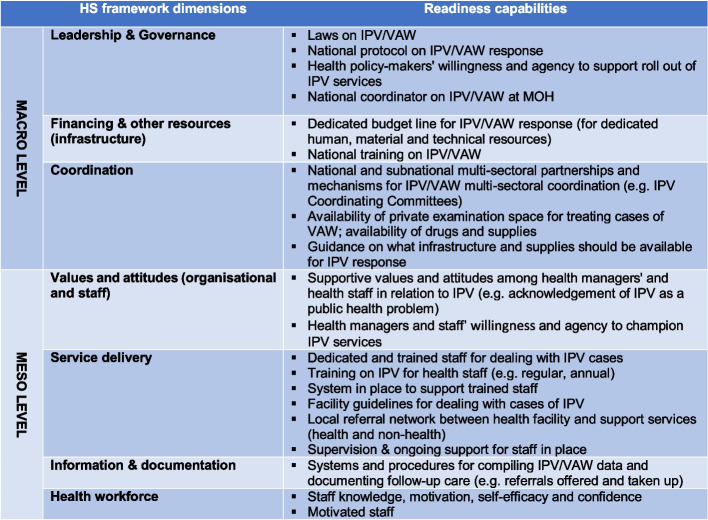
Initial conceptual framework for assessing health systems readiness to integrate IPV response. Legend: IPV = intimate partner violence; MOH = Ministry of Health; VAW = violence against women

Extending previous work based on the WHO building blocks [15, 17, 28], we developed a conceptual framework focusing on core system readiness capabilities (processes and conditions enabling health systems to be ready to adopt and implement a new intervention). The development of the initial framework occurred in two phases. First, a rapid review of studies on health system barriers and enablers to integrating IPV care [10, 11, 14, 29, 30] and on readiness and health systems strengthening [10, 16, 19, 28, 30–34] was undertaken by MC (and SM) to identify capabilities for the framework. Following the review, the framework’s key dimensions relating to health systems readiness and assessment were identified and described. The initial framework – as represented in Fig. 1 below - consisted of seven key health systems dimensions: six based on mainstream health system frameworks (governance and leadership; resources and infrastructure; information and documentation; service delivery; health workforce and coordination) [15, 35] and one cross-cutting dimension of *values* [36]. It focused on both macro (national and subnational levels) and meso/facility-level factors and their interconnections to assess the material capacity of the designated health facilities and understand operational readiness at organisational/facility levels.

The framework was organised around two levels of analysis: macro (structural) and meso (organisational - functioning of the organisation - and service delivery and health workforce) levels. The framework identified overarching elements of core system readiness capabilities (processes and conditions enabling health systems to be ready to adopt and implement a new intervention) for each of these two levels. The macro level of the framework focuses on structural capabilities, such as a clear policy framework and governance structure (i.e., accountability mechanisms), which affect how integrated care for IPV can be delivered. A key question at this level is whether laws and policies are available to support and guide an IPV health system response. The existence of strong governance (e.g. IPV law) is critical, but not always a guarantee for legitimizing (and financing) interventions and the work of motivated providers [37, 38]. For a health system to be ready to integrate IPV services, evidence shows that supportive leadership, including political will from high-level policy-makers and health managers, and tangible support, including financing for training staff, information systems, and service infrastructure, are critical elements that also influence other capabilities [14].

Coordination at national and subnational levels within health and across sectors (either through inter-agency committees or multi-sectoral teams) was also found to be essential because IPV response requires ongoing support from a range of sectors and organisations and an increased understanding and trust between organisations about their respective roles [39–41].

The meso level of the framework considers organisational, service delivery and provider-level capabilities which encompass organisational culture, structures, resources, but also processes and continuous interactions across structures and levels that are necessary for integration of IPV services and that lead to change. The key questions here are whether the organisation and the health services are sufficiently functional to deliver IPV care, and if health providers are prepared to respond to IPV cases. Having sufficient technical elements (often affected by macro-level capabilities), including resources - human, financial and material – clear protocols, a private space, adequate supplies, training and mentoring/support systems, and referral networks is necessary to enable IPV care [15]. For instance, it is known that linkages between referral structures (within health and across sectors) facilitate a comprehensive response to IPV [42, 43]. However, other issues are essential in shaping individual provider readiness, and particularly the agency and motivation of health providers. Although training of all staff is an enabling factor for implementing IPV care [14], it is not sufficient without staff motivation and self-efficacy [9]. Addressing staff values and attitudes around IPV and harnessing support and commitment from health managers are also necessary to ensure a conducive and enabling environment [14, 20, 38, 42], especially when paired with supportive interdisciplinary teams [10, 37].

## Methods

During the second phase of the development, the initial draft framework was reviewed and discussed by researchers of the Healthcare Responding to Violence and Abuse (HERA) study – a mixed-method intervention study that aimed to strengthen the primary care response to IPV in Brazil and Palestine (including all co-authors and 1 advisor [SM]) [44]. This was to determine if the framework was practical and understandable, and to discuss whether it was necessary to remove or add any element considered redundant or missing. The initial framework’s capabilities guided the development of related data collection tools (including topic guides for qualitative interviews and checklist for facility observations) for exploring health system readiness – one of the HERA study aims - in the formative phase of the study.

During this phase of the framework development, primary data collected from Brazil and Palestine between June and December 2017 (as part of the HERA study) were analysed using the framework’s dimensions to explore its appropriateness for capturing readiness capabilities. Multiple qualitative data sources (explained in Table 1 – also for each Phase of the framework development) included 43 qualitative interviews with key health stakeholders (Brazil = 16 providers and 4 managers; oPT = 10 providers, 2 managers and 11 key informants) and 4 facility observations (2 per country). The methodology and the country specific data analysis is reported elsewhere [13, 45]. Data analysis of the formative data was synthesized and revealed some gaps in the appropriateness of the framework to identify readiness capabilities. Subsequently, there followed a third phase in which the framework was adapted and refined to include issues that were missing.

**Table 1:** Description of multiple data sources and contribution to readiness analysis

Phases of the development of the framework	Data source	Description	Contribution to readiness analysis
Phase 1. Initial development	Literature	▪ Rapid literature review of studies on health system barriers and enablers to integrating IPV care and on readiness elements	▪ Identification of system capabilities for the development of the initial conceptual framework
Phase 2: Examination of appropriateness of the framework’s dimensions	In-depth interviews with health providers	▪ Views and attitudes among health providers on IPV response; knowledge, ability to offer IPV care; training received; perceived challenges; supervision and ongoing support;	▪ Values and organisational culture ▪ Health provider preparedness
	In-depth interviews with health managers	▪ Views and beliefs about IPV response and role of health systems; views on role of managers in supporting clinical team; monitoring and evaluation; allocation of resources; views on challenges	▪ Values ▪ Organisational support and leadership ▪ Management at operational level
	Facility observations	▪ Data on available resources, including availability of protocols and guidance documents, dedicated staff, adequacy of infrastructure (including confidential space to discuss IPV); availability of referral mechanisms and multi-sectoral coordination; documentation process	▪ Organisational and service delivery capabilities (facility readiness)
	Document review	▪ Document analysis of national policy documents, guidelines and reports on IPV	▪ Governance and leadership ▪ Monitoring & Evaluation/surveillance
Phase 3: Refinement of the initial framework	Data synthesis		

This paper presents the results of this refinement process, drawing on examples from the primary data to illustrate and explain the framework dimensions, its application and its refinements.

## Results

### Appropriateness of the framework

Using the framework’s dimensions to analyse HERA primary data from Brazil and oPT highlighted deficiencies in policy and practice that needed to be addressed to implement an effective response. The framework helped anticipate readiness gaps and understand system readiness capabilities and pre-conditions that were critical for integrating the HERA intervention within the existing health facilities. Table 2 below summarises the key country findings from the 43 qualitative interviews (based on the initial framework dimensions). Detailed country summaries are reported elsewhere [13, 45].

**Table 2:**
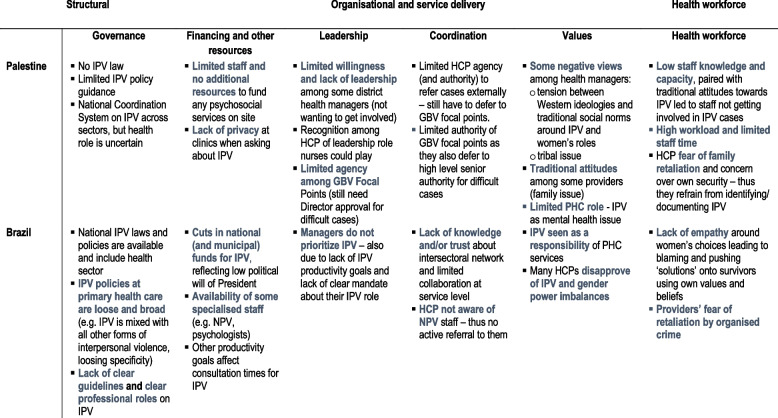
Summary of key findings of health systems readiness assessment in Brazil and Palestine

*Abbreviations: IPV* Intimate partner violence, *HCP* Health care providers, *GBV Gender-based violence*, *NPV* Violence Prevention Nucleus (NPV), *PHC* Primary health care

At macro-level, we found that governance did not automatically translate into effective implementation. In Brazil, the lack of a consistent and clear policy on IPV in primary health care (e.g. regarding provider roles, monitoring and client flows) and accountability structures affected IPV service implementation, as health providers and managers felt less confident in responding to IPV survivors [46]. Lack of training on IPV – showing lack of political will from top health managers - also affected providers’ knowledge and efficacy to work on IPV.

Additionally, the results of the data synthesis illustrated the importance of governance-related issues such as having political will, which in turn influenced the availability of clear guidance on roles and responsibilities for both health managers and clinicians, and for coordination. In both settings, it also validated the need to address the lack of perceived management support in the facility environment (evidenced by lack of training and support offered to providers), which impacted on providers’ confidence in responding to violence (and ultimately on their knowledge). The testing of the framework reiterated the vital influence of ‘software’ issues of the health system (e.g. values, leadership, support, power dynamics, relations) upon which collective readiness and commitment are contingent.

For both contexts, improving governance and capacity of the health workforce (IPV awareness, safety) to respond to IPV, were important elements that needed to be strengthened prior to adopting the new intervention. Furthermore, to overcome the lack of clarity around roles and the limited coordination, IPV specialists (Gender Based Violence (GBV) focal points in oPT and ‘Nucleus’ of Violence Prevention (NPV) staff in Brazil) participated in the initial training sessions of the pilot intervention.

In Brazil, although almost all health managers had limited knowledge and contact with IPV specialized legal and psychosocial services, personal contact and direct communication between social workers and external agencies boosted multi-sectoral collaboration [45]. Conversely, in Palestine, lack of clarity and specificity around roles and responsibilities across different healthcare providers and external services hampered collaboration [13]. The examples presented here clearly demonstrated the connections between capabilities across macro and meso levels.

### Further refinements and finalisation of the readiness framework after analysis of Brazil and oPT data

Five themes emerging from the data synthesis led to refinements of the initial framework.

First, providers needed health managers and institutional support to respond to IPV. The importance of enlisting support and commitment from health managers was evident in Brazil and Palestine, where having committed IPV leaders (e.g., regional health managers in Brazil or GBV Focal Points at Ministry of Health in Palestine) was crucial for supporting the introduction – and continuous support - of new services [13, 45]. The importance of a supportive organisational environment was thus strengthened in our framework as a key readiness element, particularly in relation to having supportive managers, supportive teams and a supportive organisational culture that legitimises the key role of providers in IPV care and not condoning IPV. This capability was made more evident in the final framework.

**Fig. 2:**
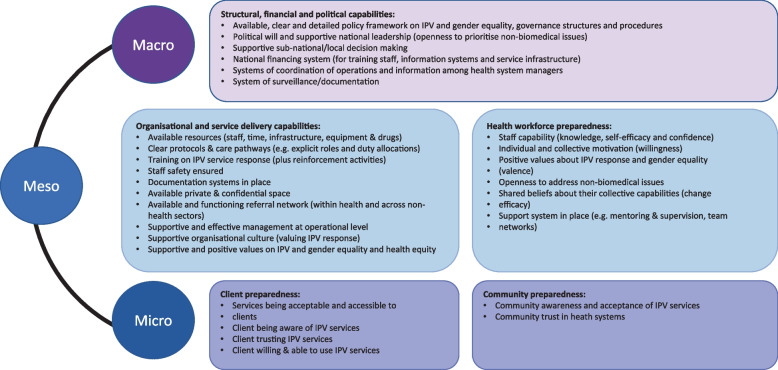
Final conceptual framework for assessing health systems readiness to integrate IPV response

Second, the importance of openness to prioritise non-biomedical issues, which emerged as an important element for readiness, particularly in Brazil. IPV was less prioritised in relation to other health issues, especially when no physical injuries were reported. Whilst some providers’ narratives highlighted IPV as unacceptable (especially in Brazil), many still thought it was not part of their role (but more relevant for mental health specialists) and had expectations of “fixing” DV by pressuring the woman to denounce the abuse. Our findings, particularly the ones from Brazil, have demonstrated how an openness to consider and prioritise problems that are not usually defined as health issues from a traditional biomedical view is also important to IPV readiness at both macro and meso levels. This can help ensure there is political will to recognise and address IPV as a health priority. This capability was subsequently added to the revised conceptual framework (see Fig. 2) at both macro and meso levels (provider’s readiness).

Third, women’s distrust in health providers’ responses and community stigma emerged as barriers to readiness. In Palestine, most women lacked trust in disclosing IPV to health providers and in their ability to maintain confidentiality [47]. Community stigma resulted in women’s concealment of IPV and providers’ fear of getting involved proved to be a barrier to identifying and documenting cases of IPV survivors at the facility [13]. Two major capabilities that emerged from the data synthesis were also services being acceptable and accessible to clients, and community stigma hindering support and engagement. Beyond trust, acceptability also entailed whether the services were responsive to women’s needs and what the women and communities really wanted and expected. In Brazil, community health workers were seen as significant for IPV detection (and at promoting dialogue with the community on IPV), but often women asked them to respect their confidentiality by not reporting IPV to the clinic/facility for fear of stigma and retaliation [48].

Fourth, survivors’ and communities’ interactions and perceptions of VAW services were also shaped by traditional beliefs around IPV and gender roles [49]. This was apparent in our data synthesis in both countries, and particularly in Palestine. Women’s limited uptake of external referral services – due to fear of stigma and retaliation - was a finding in the formative (oPT) and evaluation phases of the HERA intervention in both countries. To address the limited IPV disclosure and uptake of IPV services by women, general information sessions were organised only with women using services (and not men) in oPT [13]. Following the data synthesis, a new ‘micro’ level dimension on women and community engagement was subsequently added when refining the framework.

Last, gender values and power inequalities within the health systems affected its readiness to respond to IPV. Although the application of the initial framework considered the influence of cultural and social norms on providers’ attitudes, values and motivation, we did not use a gender equality and intersectional lens in the analysis of the readiness data during our HERA pilot. We treated health providers and managers as homogeneous groups, without considering sex, gender relations and other social markers (e.g. class and race) that may have also affected their values, preparedness and their role as health care providers (in relation to IPV). However, our data showed instances where gender norms impacted on how health providers responded to IPV. For example, victim blaming emerged as a common barrier among health providers, in both countries, particularly among Palestinian male doctors. Additionally, in Brazil, the role of community health agents - often female and from the same community as some of the clients – was not fully explored. They were perceived to have less power (by other providers) within facilities in comparison to other health providers, but appeared to instil more trust from women in the community. Furthermore, in Brazil we overlooked the importance of personal experiences of violence among female health workers (which appeared strongly during the implementation of the intervention). It is important to acknowledge that health providers usually mirror the values and experiences of the communities they come from and many female health providers may be being subjected to IPV (and other forms of abuse) themselves (or condoning it). Therefore, when finalising the framework after the testing, it was decided to make gender equality and intersectional issues more visible across all levels, particularly in relation to organisation and heath workforce capabilities (meso) and structural ones (macro). A gender and intersectional analysis could have highlighted differences in values and beliefs between male and female providers and managers in Palestine that would have better informed the content of our intervention.

The final framework for assessing health system readiness to offer care for survivors of IPV is presented in Fig. 2.

### Application of the framework

The framework also informed adaptation of the intervention. Framework dimensions and emerging findings from the readiness analysis were discussed and validated during a meeting with HERA researchers (and co-authors). Findings from the formative phase of HERA were also presented at local participatory stakeholders’ workshops with health-policy makers, district health managers, NGOs and other key informants in each country. Stakeholders discussed adaptations to the proposed HERA intervention model that would be required to support the delivery of quality care for survivors of IPV through the HERA intervention. Readiness findings pointed to concrete actions and requisites in each system dimension important for delivering the intervention effectively and helped identify assumptions for the intervention Theory of Change (ToC). Table 3 shows examples of how the readiness findings were used to inform adaptations to the HERA intervention in Brazil [45] and oPT [13].

**Table 3:** Country adaptations of the HERA intervention according to key readiness gaps

Building blocks	Key barriers affecting systems readiness		Adaptations to DV intervention content	
	Brazil	oPT	Brazil	oPT
**Governance & leadership**	IPV policies are loose and broad	Lack of clear guidance on IPV	• Provide clear information on guidelines and referral flow during training • Develop and disseminate leaflets detailing Standard Operating Procedures (SOPs) and care flows	• A simplified brief clinic guide book with referral pathway and roles was developed jointly with MOH
	Managers do not prioritize IPV		• Include/invite managers in the IPV training • Discuss how to consider IPV as an indicator in consultative committee	
**Health workforce and service delivery**	Lack of clear professional roles	Low staff knowledge and capacity on DV Lack of clarity of roles	• Intervention clarifies roles for all health professionals, managers and NPV teams	• Training intervention to raise IPV awareness of all clinical staff not just nurses • MOH GBV focal points attended initial training sessions to clarify roles
	Lack of clear protocol and flows		• Establish an agreed protocol and flow based on current policies and international evidence.	
	Lack of empathy around women’s choices leading to blaming and pushing ‘solutions’ onto survivors using own values and beliefs		• Use interactive game (‘In your shoes’ Brazilian version) and role plays to ensure HCPs	
	Providers fear of family retaliation		• Discuss confidentiality, safety plan for women and providers (including home visits), and clarify manager’ support in relation to safety	• Integration of discussion on staff security in the training content
**Coordination**	Lack of knowledge about intersectoral network and limited collaboration	Limited knowledge of referral services	• Organize introductory meetings between specialized services and NPV teams	

*Abbreviations*: *HCP* Health care providers, *GBV* Gender-based violence, *NPV* Violence Prevention Nucleus (NPV), which is responsible for coordinating care in cases of IPV within the PHC Clinic in Brazil, *PHC* Primary health care

Although it was beyond the scope of the HERA intervention to address readiness gaps across all system levels, especially at macro level, having an understanding of the structural and contextual factors that could affect successful implementation of the intervention proved useful during the development of the ToC and the evaluation phase [50]. Evaluation findings from oPT show how some readiness gaps still persisted post-implementation, such as providers’ fear for their own safety and lack of management support [50].

## Discussion

The proposed framework identifies: a) what system capabilities and pre-conditions are needed for a health system to be ready to provide IPV care; b) what system changes are required to deliver quality care for survivors of IPV; and c) what connections exist between and across systems’ levels and capabilities and where positive interactions and bottlenecks may be. In particular, the application of our framework to our primary data enabled us to detect anticipated preparedness gaps within different health system dimensions and allowed a nuanced analysis of the interactions between them.

The innovation of this framework lies in its systemic approach to readiness - by merging and adapting constructs from scholarship on organisational [22, 51], service [15, 52], and provider [19, 53, 54] readiness for change. Our framework recognises the centrality of human and institutional relationships, ideas and interests, values and norms, affinities and power dynamics. These are as important as the more tangible resources commonly measured such as organisational policy, legal and material resources and structures such as infrastructure, supplies, management information systems, and financing [55].

While measuring indicators that are rapidly observed by patients seeking care – e.g. staff attitudes and waiting times – can be helpful, it is important to study how upstream factors, such as supportive management practices (e.g. prioritising discussion of IPV in regular meetings, monitoring implementation of IPV services and offering feedback to providers; allowing providers to attend IPV training) matter, for example by influencing health providers’ morale and confidence, and by creating a positive implementation climate. The latter is about the providers’ collective perception that the intervention is supported, expected and rewarded in the organisation There is increasing recognition in the health systems literature that health professionals do not act in isolation and that governance, effective and supportive management and structural factors also determine the performance of health systems and their providers [56, 57]. How health providers engage with and are supported by the health system matters because it shapes the quality of care they are able to provide, as well as how they engage with the communities they serve to promote health. Leadership of, and engagement with, senior managers in the health system is an evident organizational facilitator for integrating IPV services, especially horizontal leadership that encourages health workers [28, 37]. Despite that, evidence on the role of management practices in influencing the quality of care – especially at district or facility levels – is limited in LMICs and is mostly documented in fields other than VAW [50, 58–60]. Furthermore, health managers also operate within the system’s constraints (e.g. reduced resources, high workload, centralised decision-making, low motivation to implement IPV care; institutional norms) and within institutionally sanctioned rules that govern behaviour and structure relationships that may affect sustainability of integrated IPV care [61]. Future research should include a better understanding of managerial systems’ constraints and what leadership qualities management cadres should have and how these can be nurtured and sustained.

Our analysis of the primary data in both countries highlighted a missing level within the initial framework: the micro level, which includes elements such as women’s readiness (to engage with what it is offering as a response to IPV) and community support. A novelty of the conceptual framework is the capabilities related to the women and community engagement and the need to also consider how the system can sustainably involve and interact with clients (women) and communities to ensure and promote readiness for integrating change. Without this, other health system capabilities are weakened (as we saw for instance in our findings in relation to low women’s referral uptake [44]). The inclusion of this dimension was not sufficiently addressed in the initial WHO building blocks model [62] though subsequently mentioned in the WHO VAW management response [15]. The testing of the initial framework in Brazil and Palestine highlighted that community trust and engagement, while decisive to reducing stigma and enabling women’s access to IPV care, was limited. Key to acceptability (and therefore uptake) of VAW services is communities’ and clients’ awareness of and trust in the new services and in the providers who offer them [13, 27]. A recent review on women’s expectations after IPV disclosure also reiterated the importance emotional connection with providers and maintaining women’s autonomy in the response approach [63]. The experience of the COVID-19 pandemic has also revealed just how valuable community engagement is to build trust in health systems in all settings to ensure support, compliance to social measures and vaccine uptake. Innovative bottom-up opportunities have emerged reinstating the roles of families, communities and of NGOs in supporting the overall health of populations during COVID-19 [64, 65].

Besides clinical effectiveness, perceptions of the quality of care by clients and communities are likely to be the key drivers of utilization [58]. Patients’ trust in services has also been shown to be an important element of perceived quality [45] and responsiveness [65]. The incorporation of women’s and communities’ perspectives is crucial for identifying the needs and preferences of survivors [15], but also social and gender norms that may affect demand and uptake of VAW services. In particular, traditional views on restrictive gender roles and IPV, and stigma towards IPV are renowned barriers to help-seeking for abused women [66, 67], and have to be addressed when providing IPV care. Meso-level capabilities that address stigma and gender inequality (e.g. training opportunities addressing gender norms; skills to be able to support women through an autonomous process of change and decision-making, and staff’s ability to respect confidentiality) can positively affect client and community trust. More participatory and ethnographic research is warranted on how community engagement is related to successful implementation of IPV interventions, particularly to understand ‘trust’ and barriers to access and help-seeking behaviours and how to strengthen the linkages between community and health-based services.

Additionally, it is known that restrictive gender norms and gender inequalities are replicated and reinforced in health systems (across all levels) contributing to gender inequalities in health [68, 69]. Based on the growing recognition that gender equality/inequality is a key social stratifier in health systems [70, 71], the final framework included consideration of gender equality and also articulated its intersection with other social identities (e.g. class, age, race etc) that further contribute to inequalities in health and health care [72, 73]. Our findings pointed also to the power imbalances within the hierarchy of the health systems and how this could also impact on IPV response, especially in relation to assistant nurses and community health workers, who are often female and less influential. The inclusion of a gender and intersectional lens was a second new addition to the initially conceptualised framework, which cut across all levels. The work on VAW is pivotal for giving prominence to gender inequalities in the health system and adopting a gender lens to the analysis and ensure women’s participation in leadership, as suggested elsewhere [74, 75].

Another central feature of our framework is the importance of linkages and interactions between capabilities at each system level, which also became evident when testing the framework in Brazil and Palestine [13, 45]. The interconnection across system levels and capabilities is necessary to ensure that the focus remains systemic and recognizes the complex feedback loops between and among all the levels and factors. Rather than fall back to one level (e.g., only provider, or clinical), all readiness factors need to be assessed, and the reciprocal interactions made visible across system levels. Even when frontline providers have substantial discretion in their interpretation of regulations and freedom to adapt treatment protocols, their actions may still largely depend on upstream factors related to institutional capacity, legal sanctions and institutional norms [58]. For example, the limited guidance on IPV and the hierarchical referral system in place in Palestine limited the role and the authority of the GBV Focal Points within the Ministry of Health [13].

With a readiness lens, it is possible to design more tailored interventions that consider facilitators and barriers at different levels of the health system, and that recognise the critical importance of working closely with managers and stakeholders. In addition, our conceptual framework for the readiness assessment has proven useful in highlighting significant systemic issues for developing health system interventions and their evaluation [44].

### Limitations of the framework

We acknowledge the following limitations. First, although the framework was revised to include a micro level analysis around women’s and community engagement, it would require further testing to ensure such domains are being fully captured. Second, the same would be true for the adoption of a gender equality lens and whether it is possible to analyse gender and its intersection with other social determinants across all levels with the existing readiness capabilities.

Third, the operationalisation of the framework is complex and requires multiple sources of data collection and a multi-layered analysis. With the above caveats, we emphasize that the use of this framework allows a comprehensive analysis of readiness gaps and enablers across all levels of the health systems, which is critical for IPV interventions, even those with one-dimensional focus (on one specific systems level such as service-delivery or providers’).

Lastly, although the framework was conceived for LMIC context, however, with adaptations, it could be used in high income settings.

## Conclusion

In this paper, we present a new conceptual framework for analysing and understanding health system’s readiness for integrating IPV response into healthcare services that has been tested in two sites. A new contribution of our framework is that it captures system capabilities beyond the availability of material resources and technical capacity of providers to encompass, for instance, stakeholders’ values, confidence and motivation and their connection with clients and communities. This can inform a better understanding of how health systems can reach communities and people who need to access health services. Future research should also determine the ways in which community or organizational preparations are related to implementation success.

## Data Availability

The datasets used and/or analysed during the current study are available from the corresponding author upon reasonable request.

## Abbreviations

GBV: Gender-based violence
HCP: Health care providers
HERA: Healthcare Responding to Violence and Abuse
IPV: Intimate partner violence
LMICs: Low- and middle-income countries
MoH: Ministry of Health
NGOs: Non-governmental organisations
NPV: Violence Prevention Nucleus
oPT: Occupied Palestinian territories
PHC: Primary health care
ToC: Theory of Change
VAW: Violence against women
WHO: World Health Organization
